# GSTP1 promoter hypermethylation in prostate cancer detection: current evidence, clinical positioning, and translational gaps

**DOI:** 10.1007/s11033-026-13011-2

**Published:** 2026-10-21

**Authors:** Jun-Lynn Joo, Rebecca Shin-Yee Wong, Bey Hing Goh, Nancy Choon-Si Ng

**Affiliations:** 1 Department of Biomedical Sciences, Jeffrey Cheah Sunway Medical School, Faculty of Medical and Life Sciences, Sunway University, Sunway City, Malaysia; 2 Department of Medical Education, Jeffrey Cheah Sunway Medical School, Faculty of Medical and Life Sciences, Sunway University, Sunway City, Malaysia; 3 Faculty of Medicine, University of Cyberjaya, Cyberjaya, Malaysia; 4 Centre for Learning and Teaching, University of Cyberjaya, Cyberjaya, Malaysia; 5 School of Clinical and Health Sciences, Faculty of Health, University of Technology Sydney, NSW 2007 Sydney, Australia; 6 Sunway Biofunctional Molecules Discovery Centre, Faculty of Medical and Life Sciences, Sunway University, Sunway City, Malaysia; 7 Graduate Institute of Cancer Biology and Drug Discovery, College of Medical Science and Technology, Taipei Medical University, Taipei, Taiwan; 8 Graduate School of Medicine, KPJ Healthcare University, Nilai, Malaysia

**Keywords:** Prostate cancer, GSTP1, DNA methylation, Biomarkers, Liquid biopsy, Early detection

## Abstract

GSTP1 promoter hypermethylation is one of the most consistently documented molecular alterations in prostate cancer, with high prevalence in malignant tissue and a well-established relationship with transcriptional silencing. However, despite more than two decades of biological and diagnostic investigation, GSTP1-based testing has not acquired a clearly defined stand-alone role in contemporary prostate cancer detection. This narrative review examines why extensive biological evidence has not translated into routine clinical use and where, if anywhere, GSTP1 testing might provide meaningful value within current diagnostic pathways. Evidence from prostate tissue, urine and circulating cell-free DNA is considered alongside the effects of biospecimen selection, preanalytical handling, CpG target selection, assay design and interpretation thresholds. Tissue demonstrates the most reproducible signal and has achieved partial clinical translation through multiplex testing such as ConfirmMDx, whereas urinary and blood-based approaches remain investigational. Recent urinary findings, including in men with moderately elevated PSA, are promising but remain preliminary and have not established incremental value beyond contemporary risk assessment, biomarkers and multiparametric MRI. The translational priority is therefore not to demonstrate again that GSTP1 detects prostate cancer, but to determine whether a standardised assay provides reproducible, nonredundant and clinically actionable information at a defined decision point. Future development should prioritise clearly specified target populations, analytically validated assay configurations, clinically significant prostate cancer as the relevant endpoint, and prospective evaluation against contemporary diagnostic pathways.

## Introduction: GSTP1 methylation in contemporary prostate cancer detection

Prostate cancer remains a major contributor to cancer incidence and mortality among men worldwide. More than 1.46 million new cases and approximately 396,000 deaths were estimated globally in 2022, with substantial geographical variation [1, 2]. Incidence is particularly high in regions with extensive prostate-specific antigen (PSA) testing, whereas mortality remains disproportionately elevated where access to timely diagnosis and treatment is more limited [2]. These patterns highlight a fundamental challenge: increasing cancer detection is not necessarily equivalent to improving detection of cancers for which earlier diagnosis is clinically consequential.

Contemporary prostate cancer detection has moved beyond a simple pathway of elevated PSA followed directly by biopsy. PSA remains central to initial risk assessment, but its limited specificity is well recognised. In symptomatic populations, Merriel et al. reported pooled sensitivity of 0.93 but specificity of only 0.20 for a PSA threshold of ≥ 4 ng/mL for detection of any prostate cancer [3]. Modern pathways increasingly integrate clinical risk factors, PSA derivatives, risk calculators, multiparametric magnetic resonance imaging (mpMRI), adjunctive serum or urine biomarkers, and targeted or systematic biopsy. Contemporary guidelines emphasise detection of clinically significant prostate cancer (csPCa), generally defined as Grade Group ≥ 2, rather than maximising identification of all histological cancers [4, 5].

Within this changing landscape, glutathione S-transferase pi 1 (GSTP1) promoter hypermethylation occupies an unusual position. Few molecular alterations in prostate cancer have been investigated so consistently over time. Aberrant methylation of the GSTP1 promoter CpG island was recognised more than two decades ago and subsequently shown to be highly prevalent in malignant prostate tissue while uncommon in histologically normal prostate epithelium [6, 7]. Mechanistic studies established a close relationship between promoter hypermethylation and suppression of GSTP1 transcription, while newer evidence has reinforced the early occurrence and high prevalence of this alteration during prostate carcinogenesis [8, 9].

The magnitude of this tumour-associated signal has been repeatedly demonstrated. Martignano et al. detected GSTP1 methylation in 91.1% of prostate cancer tissues compared with 5.4% of adjacent healthy tissues and demonstrated an inverse relationship between methylation and GSTP1 protein expression [10]. Pidsley et al. subsequently detected methylated GSTP1 in 97% of radical-prostatectomy tumours [11]. Such findings establish strong biological validity but do not, by themselves, establish clinical validity or utility.

The apparent attractiveness of GSTP1 extends beyond tissue. Methylated GSTP1 has been investigated in urine, plasma, serum, circulating cell-free DNA (cfDNA), prostatic secretions, and ejaculates using multiple analytical platforms [6, 12–15]. This combination of high tumour prevalence, biological plausibility, molecular stability, and compatibility with targeted assays might be expected to favour clinical translation. Nevertheless, stand-alone GSTP1 methylation testing has not acquired a clear role in routine detection.

This discrepancy constitutes the central translational problem addressed in this review. The relevant question is no longer whether GSTP1 promoter hypermethylation is associated with prostate cancer; that association is well established. Nor is it sufficient to show that an assay distinguishes selected cancers from controls under experimental conditions. The more clinically relevant question is whether measuring GSTP1 provides incremental, reproducible, and actionable information at a defined decision point within the diagnostic pathway.

This distinction matters because the clinical environment surrounding GSTP1 has changed substantially since its earliest diagnostic studies. Earlier investigations often positioned methylation testing as an adjunct to PSA or a means of reducing unnecessary biopsy. Contemporary assessment increasingly incorporates PSA density, multivariable risk estimation, mpMRI, MRI-targeted biopsy, and established blood-, urine-, and tissue-based biomarkers. The appropriate comparator for a new GSTP1-based assay is therefore no longer PSA alone, but the information already available within multimodal care.

Recent literature also makes another conventional review of GSTP1 diagnostic accuracy less informative. Broad reviews have addressed prostate cancer epigenetics and DNA methylation [8, 16, 17], while others have examined liquid-biopsy biomarkers more generally [18–20]. Meta-analyses have evaluated the diagnostic and prognostic performance of GSTP1 methylation itself [15, 21]. Most recently, Cleanclay et al. (2026) identified GSTP1 and RASSF1A as the most frequently reported liquid-biopsy DNA methylation markers, but found moderate performance for individual markers and higher accuracy with multigene strategies. These mature evidence bases permit a shift from asking whether GSTP1 is a diagnostic biomarker to asking why extensive biological evidence has produced limited clinical translation.

Unlike previous reviews that have primarily synthesised prostate cancer epigenetics, liquid-biopsy biomarkers, or the diagnostic and prognostic performance of GSTP1 methylation, this review integrates three levels of evidence: biological and clinical findings across tissue, urine and circulating cfDNA; preanalytical and assay-related determinants of measurement; and the potential role of GSTP1 at defined decision points within contemporary diagnostic pathways. This translational framework is used to examine why strong biological validity has not resulted in an established clinical role and to identify the assay configurations, target populations and clinical decisions that should be prioritised in future studies. The central issue is therefore not simply whether GSTP1 can detect prostate cancer, but whether it provides reproducible, incremental and clinically actionable information beyond contemporary multimodal care.

### Literature search and review approach

This narrative review was informed by a targeted literature search in PubMed from database inception through August 2026. Search terms combined concepts related to “GSTP1”, “prostate cancer”, “DNA methylation”, “promoter hypermethylation”, “urine”, “cell-free DNA”, “liquid biopsy”, “diagnosis”, “prognosis”, “biomarker”, and “magnetic resonance imaging”. The literature considered included primary studies evaluating GSTP1 methylation in prostate tissue, urine, plasma, serum, or circulating cell-free DNA; systematic reviews and meta-analyses; studies addressing preanalytical or analytical factors affecting methylation measurement; and contemporary guidelines or reviews relevant to prostate cancer diagnostic pathways. Studies were selected according to their relevance to the biological validity, analytical performance, clinical validity, clinical utility, or potential clinical positioning of GSTP1 methylation. Additional references were identified by screening the reference lists of key articles. As this was a narrative rather than a systematic review, no formal meta-analysis or risk-of-bias assessment was undertaken.

## Biological basis of GSTP1 promoter hypermethylation in prostate carcinogenesis

DNA methylation is one of the best-characterised epigenetic mechanisms in malignancy. Methyl groups are added predominantly to cytosine residues within CpG dinucleotides, and methylation within promoter-associated CpG islands can alter transcriptional activity. In cancer, focal promoter hypermethylation can contribute to stable transcriptional repression, whereas broader epigenomic changes accompany genomic instability, tumour evolution, and treatment resistance [8, 16, 17]. In prostate cancer, these alterations occur across numerous loci, including GSTP1, APC, RASSF1A, and RARB, within an epigenetic landscape that evolves from early neoplasia through advanced and treatment-resistant disease. Recent reviews emphasise that epigenetic alterations can arise early in the natural history of prostate cancer, including in precursor lesions, although only a minority have progressed towards clinical implementation [8].

GSTP1 encodes the pi-class glutathione S-transferase, an enzyme involved in glutathione conjugation of electrophilic compounds and protection against oxidative and genotoxic stress. GSTP1 also participates in signalling pathways regulating proliferation, apoptosis, and cellular stress responses [22]. Its expression pattern during prostate carcinogenesis is notable: GSTP1 is highly expressed in normal basal cells, expressed at low levels in normal luminal epithelium, and is often induced in proliferative inflammatory atrophy (PIA), potentially as a response to chemical or oxidative stress; by contrast, GSTP1 expression is frequently lost in high-grade prostatic intraepithelial neoplasia (HGPIN) and prostate adenocarcinoma in association with promoter CpG island hypermethylation [9]. Loss of this protective activity may increase the susceptibility of prostatic epithelial cells to additional molecular injury [6].

The causal relationship between promoter methylation and transcriptional suppression is supported experimentally. Lin et al. (2001) demonstrated GSTP1 CpG island hypermethylation in 40 of 42 prostate cancers, while corresponding methylation was absent from normal prostate specimens. In LNCaP prostate cancer cells, treatment with the DNA methyltransferase inhibitor 5-azacytidine reversed promoter hypermethylation and restored both GSTP1 transcription and GSTP1 protein expression [7]. Experimental methylation of GSTP1 promoter constructs likewise reduced promoter activity, providing direct evidence that CpG methylation can silence GSTP1 transcription.

Nevertheless, GSTP1 should not be described simply as a conventional tumour-suppressor gene. In the same study, restoration of GSTP1 expression did not materially suppress the growth or tumourigenicity of already transformed LNCaP cells [7]. The investigators therefore proposed that GSTP1 loss may facilitate susceptibility to subsequent molecular damage rather than functioning as a classical gatekeeper whose restoration reverses the malignant phenotype. This distinction is important for biomarker interpretation: GSTP1 methylation is highly characteristic of prostate malignancy, but its presence or abundance should not automatically be interpreted as a direct measure of tumour aggressiveness.

Contemporary primary evidence provides a more detailed view of when GSTP1 methylation emerges. Gupta et al. (2023) used bisulfite genomic sequencing of laser-capture microdissected normal epithelium, PIA, HGPIN, and prostate adenocarcinoma from 32 radical-prostatectomy specimens. Only 0.52% and 1.3% of evaluated CpG sites were methylated in normal epithelium and PIA, respectively, compared with 24% in HGPIN and 51% in invasive carcinoma. HGPIN therefore showed an intermediate methylation density between largely unmethylated normal/PIA tissue and more densely methylated cancer. Methylation was preferentially concentrated around the transcription start site and AP1/SP1 transcription-factor binding regions, supporting a progressive “seeding and spreading” model across the GSTP1 promoter during neoplastic progression [9].

This progressive acquisition helps explain the strong diagnostic signal in established carcinoma. Martignano et al. (2016) detected GSTP1 methylation in 51 of 56 malignant specimens and showed that all methylated cancers lacked corresponding GSTP1 expression [10]. Pidsley et al. (2024) similarly detected methylated GSTP1 in 97% of radical-prostatectomy tumours, while The Cancer Genome Atlas (TCGA) prostate adenocarcinoma data showed promoter methylation in more than 85% of tumours with accompanying suppression of GSTP1 expression [11].

However, an alteration that emerges early and becomes widespread during tumour development may carry less information about subsequent biological divergence. Pidsley et al. (2024) found associations between higher tumour GSTP1 methylation and biochemical recurrence and metastatic relapse in univariable analyses, but methylation did not retain independent prognostic value after established clinicopathological factors were incorporated into multivariable models. Thus, GSTP1 methylation may partly reflect malignant epithelial-cell content or tumour burden rather than independently capturing aggressive biological behaviour.

The GSTP1 methylation state is also more complex than a binary “methylated versus unmethylated” classification. Gupta et al. demonstrated substantial allele-level heterogeneity within HGPIN and prostate cancer, including partially methylated promoter alleles and different methylation patterns among separate tumour foci from the same patient [9]. Visser et al. subsequently identified heterogeneous GSTP1 epialleles in tissue and urine, reinforcing that different combinations of methylated and unmethylated CpG sites can coexist [23]. The biological significance of these patterns remains incompletely defined, but they indicate that the molecular state captured by a GSTP1 assay depends partly on which CpG sites are interrogated.

The biological foundation for GSTP1 as a prostate cancer marker is therefore strong but not simple. Its progressive and frequent epigenetic silencing provides a biologically coherent tumour-associated signal, whereas locus-level heterogeneity and limited independent prognostic value constrain interpretations based solely on presence or quantity. Translational development must consequently distinguish whether methylated GSTP1 identifies malignant prostate-derived DNA, whether its level or pattern identifies clinically important disease, and whether measuring it ultimately improves a clinical decision.

## From molecular alteration to measurable biomarker: assays and biospecimens

The apparent performance of GSTP1 methylation cannot be interpreted independently of the biospecimen and analytical method used to measure it. Over more than two decades, detection technologies have progressed from qualitative methylation-specific polymerase chain reaction (MSP) to quantitative MSP (qMSP), digital PCR, targeted methylation sequencing and single-molecule approaches. Contemporary evidence increasingly emphasises that biomarker performance reflects the complete measurement pathway, encompassing specimen collection and processing, DNA conversion, CpG selection, analytical platform and interpretation threshold, rather than the molecular alteration alone [24, 25].

### Tissue

Tumour tissue represents the analytically most favourable specimen because the proportion of tumour-derived DNA is substantially greater than in circulating or urinary samples. The high prevalence of GSTP1 hypermethylation observed in prostatectomy specimens therefore reflects measurement under relatively high-signal conditions and does not necessarily predict the sensitivity achievable in non-invasive samples.

Early tissue studies established a strong malignant–benign contrast. Lin et al. (2001) detected promoter hypermethylation in approximately 95% of prostate cancers while normal prostate DNA remained unmethylated [7]. Martignano et al. (2016) subsequently reported methylation in 91.1% of tumour tissues compared with 5.4% of adjacent healthy tissues [10], while Pidsley et al. (2024) detected methylated GSTP1 in 97% of radical-prostatectomy tumours [11]. These reproducible findings explain why tissue remains the matrix in which the GSTP1 signal appears most consistent.

Tissue analysis also underlies the most clinically mature application of GSTP1 methylation. ConfirmMDx measures GSTP1, APC, and RASSF1 methylation in histologically negative prostate biopsy tissue to identify an epigenetic field effect associated with potentially unsampled cancer. The American Urological Association/Society of Urologic Oncology (AUA/SUO) guideline lists ConfirmMDx among available tissue biomarker assays in the repeat-biopsy setting [5]. Thus, GSTP1 has achieved partial clinical translation, although within a defined multiplex tissue assay rather than as a stand-alone non-invasive test.

### Urine

Urine is attractive for prostate cancer biomarker development because prostate-derived cells and nucleic acids can enter the urethra and subsequently be recovered from voided urine. As a local liquid-biopsy source, it may provide a higher concentration of anatomically proximal tumour-derived material than blood. However, prostate tumours do not directly contact urine in the same manner as bladder tumours, and the amount of tumour-derived material shed into urine may remain limited [25].

Importantly, urine is not a uniform analytical matrix. Studies may analyse cellular material, urine supernatant, urinary cell-free DNA (cfDNA), or combinations thereof. Urinary cfDNA also differs from plasma cfDNA in fragment characteristics; most urinary cfDNA fragments are shorter than 100 bp, whereas plasma cfDNA typically peaks around 160–167 bp, meaning that extraction methods optimised for plasma may not efficiently recover urinary fragments [24].

Collection relative to prostatic manipulation is another major determinant of signal. Digital rectal examination (DRE) or prostate massage can increase the release of prostate-derived cells and DNA into first-void urine. Cairns et al. (2001) established the feasibility of urinary GSTP1 detection [26], whereas work summarised by Henrique and Jerónimo (2004) demonstrated substantial differences according to collection strategy [6]. Goessl et al. (2000), for example, detected methylated GSTP1 in 94% of tumour tissues but only 36% of urine samples, with higher detection subsequently reported following prostate massage [13].

Rouprêt et al. (2007) detected urinary GSTP1 methylation in 83.2% of patients with localised prostate cancer after prostate massage. A four-gene panel incorporating GSTP1, RASSF1A, RARβ2, and APC achieved a theoretical sensitivity of 86% and diagnostic accuracy of 89% [14], illustrating both the importance of prostate-enriched urine collection and the potential advantage of multiple methylation loci.

More recently, Ji et al. (2025) employed a standardised 20-second DRE followed by collection of the first 5–10 mL of urine into preservative-containing tubes before automated GSTP1 analysis [27]. The relatively strong diagnostic performance obtained suggests that improvements over earlier studies may partly reflect optimisation of collection, preservation, DNA recovery, and analytical workflow rather than changes in the underlying biomarker biology.

Preanalytical standardisation remains critical. Peng et al. (2024) identified substantial variation in urine collection, preservatives, processing delays, centrifugation, extraction, and storage, with urinary cfDNA particularly susceptible to temperature, pH, degradation, and loss of short fragments [24]. Such variation can alter the amount and integrity of recoverable DNA before methylation analysis is performed.

### Blood-based specimens: plasma and serum

Blood-based testing offers practical advantages, including familiar venepuncture and repeated longitudinal sampling, but localised prostate cancer presents a major biological limitation: low abundance of tumour-derived DNA in circulation. Circulating tumour DNA (ctDNA) constitutes only a fraction of total cfDNA and may represent approximately 1% or less in localised disease, whereas substantially greater fractions may occur in metastatic cancer [20].

For methylation detection, the relevant analytical quantity is therefore not total cfDNA but the proportion derived from tumour. Pharo et al. (2025) emphasise that assay sensitivity declines at low ctDNA fractions because the cancer-derived signal becomes increasingly diluted within DNA originating from normal tissues [25]. Rendek et al. similarly identify low DNA concentration, fragmentation, and high background noise as persistent challenges for cfDNA methylation analysis [28].

This distinction limits extrapolation from advanced disease. Fonseca et al. (2024) demonstrated substantial prognostic value of plasma ctDNA fraction in 491 patients with metastatic castration-resistant prostate cancer, but these findings do not establish equivalent analytical performance in men undergoing evaluation for localised disease [29].

Plasma and serum should also not be treated as interchangeable. Although serum may contain more total cfDNA, plasma generally has less contamination from genomic DNA released by lysed blood cells and may provide a more favourable ctDNA fraction and stability [25]. Collection tubes, processing delays, centrifugation, storage, and DNA extraction further influence cfDNA quantity and integrity [24].

These factors help explain the recurrent pattern reported for circulating GSTP1: relatively high specificity but more modest sensitivity. Detection of a cancer-associated methylated fragment may be informative, whereas failure to detect one cannot reliably exclude a small localised tumour.

### Analytical platforms

Traditional MSP generally relies on sodium bisulfite conversion, in which unmethylated cytosines are chemically converted while methylated cytosines remain distinguishable, followed by amplification with methylation-specific primers [30, 31]. The method is accessible and sensitive but depends strongly on conversion efficiency, primer design, CpG selection, and the criteria used to define a positive result [28].

Bisulfite conversion itself creates an important trade-off. Although it remains the standard pretreatment for many PCR-based methylation assays, it is chemically harsh and can substantially degrade DNA, which is particularly problematic when liquid-biopsy targets are already scarce and fragmented. Enzymatic conversion using TET2 and APOBEC-based approaches can preserve longer DNA fragments, although lower overall cfDNA recovery has also been reported. Consequently, bisulfite conversion remains practical for PCR-based assays, whereas enzymatic approaches may be particularly attractive for sequencing applications requiring greater preservation of DNA integrity [25].

qMSP provides quantitative rather than purely qualitative measurement but does not eliminate locus-level methylation heterogeneity. Visser et al. (2026) demonstrated that the location of unmethylated CpG sites within GSTP1 primer and probe binding regions altered amplification efficiency, fluorescence, and measured Cp values [23]. Prostate tissue predominantly contained fully methylated or unmethylated fragments, whereas urine contained a greater proportion of partially methylated epialleles. A qMSP assay designed around fully methylated templates may therefore underestimate partially methylated molecules and generate unstable or false-negative measurements.

Digital PCR offers advantages for rare targets by partitioning DNA molecules into numerous independent reactions and enabling highly sensitive absolute quantification. Pharo et al. (2025) note that digital PCR can achieve substantially lower analytical limits of detection than conventional quantitative PCR (qPCR) [25]. However, it remains locus-specific and therefore continues to depend on appropriate CpG selection, primer/probe architecture, and classification of positive partitions.

Sequencing provides greater molecular resolution by interrogating methylation across multiple CpGs or individual molecules. Bryzgunova et al. (2021) used locus-specific next-generation sequencing to characterise GSTP1, RNF219, and KIAA1539 methylation at single-molecule resolution in plasma cfDNA. Combinations of methylation patterns appeared to improve discrimination, although the small cohort (i.e., 20 prostate cancer cases, 17 patients with benign prostatic hyperplasia, and 18 healthy donors) raised the possibility of model overfitting [12].

Emerging enzymatic methyl-sequencing and third-generation technologies such as nanopore sequencing can detect methylation while reducing DNA damage associated with bisulfite treatment [25]. Their greater molecular resolution, however, comes with increased sequencing, bioinformatic, quality-control, and cost requirements. Targeted PCR-based platforms may therefore remain more practical for clinical validation where a small number of predefined loci are being measured.

Taken together, “GSTP1 methylation” is not a single standardised laboratory measurement. Studies may interrogate different biological matrices, CpG positions, and methylation patterns using different extraction methods, conversion chemistries, analytical platforms, normalisation approaches, and positivity thresholds. Rendek et al. (2024) further emphasise that different research groups frequently apply different criteria for defining positive and negative results and different procedures for cfDNA extraction and quantification [28].

Analytical heterogeneity is therefore not merely statistical variation between studies; it can determine which cancer-associated molecules are detected. For clinical translation, GSTP1 should consequently be regarded as part of a complete measurement system encompassing biospecimen source, collection and preservation, DNA extraction, methylation conversion, CpG target selection, analytical platform, and interpretation threshold. Standardisation across this pathway is necessary to determine whether differences in reported clinical performance reflect the biology of GSTP1 or the way in which it has been measured [24, 25].

## Contemporary evidence for diagnostic and prognostic performance

### Tissue evidence: strong diagnostic discrimination, limited incremental prognostic value

The most consistent evidence for GSTP1 derives from prostate tissue. Across foundational and contemporary studies, the biomarker demonstrates a marked separation between malignant and non-malignant tissue. Lin et al. reported promoter hypermethylation in 40 of 42 prostate cancers [7], while Martignano et al. detected methylation in 91.1% of tumours compared with 5.4% of adjacent healthy tissue, together with loss of GSTP1 protein expression in methylated cancers [10]. More recently, Pidsley et al. detected methylated GSTP1 in 97% of radical-prostatectomy tumours [11]. Representative evidence for GSTP1 methylation across tissue and liquid-biopsy specimens, including key diagnostic findings and translational limitations, is summarised in Table 1.

**Table 1:** Comparative Characteristics and Representative Evidence for GSTP1 Methylation Across Tissue, Urine and Blood-Based Specimens

Biospecimen	Evidence	Analytical advantages	Principal analytical / preanalytical limitations	Current level of clinical evidence	Translational interpretation
Prostate tissue	• Lin et al. (2001): methylation in 40/42 cancers; Martignano et al. (2016): 91.1% of tumours vs. 5.4% of adjacent healthy tissue • Pidsley et al. (2024): methylated GSTP1 detected in 97% of prostatectomy tumours	• High tumour-derived DNA abundance • Strong malignant–benign contrast • Reproducible detection across studies	• Requires invasive tissue acquisition • Tissue findings do not directly predict performance in non-invasive specimens • Prognostic value is not independently established	• Strongest evidence for biological validity and tissue-level clinical validity • Partial clinical translation through multiplex tissue testing such as ConfirmMDx	Well-established tumour-associated marker in tissue, but not a stand-alone diagnostic test
Urine	• Rouprêt et al. (2007): urinary GSTP1 detected in 83.2% after prostate massage • Ji et al. (2025): sensitivity 81.4%, specificity 83.6%, AUC 0.87; PSA 4–10 ng/mL subgroup AUC 0.90	• Non-invasive • Anatomically proximal to the prostate • Prostate-derived material can be enriched by DRE or prostate massage	• Strongly dependent on collection method, urine fraction, preservation, processing, DNA recovery, CpG target, and assay design • Heterogeneous methylation may affect qMSP performance	• Promising but heterogeneous • Strongest recent evidence remains retrospective and single-centre • csPCa-specific prospective validation is lacking	Promising non-invasive specimen for further study, but not clinically validated for routine use
Blood / plasma / serum cfDNA	• Wu et al. (2011): pooled sensitivity 0.52 and specificity 0.89 across body fluid studies • Ye et al. (2023): cfDNA sensitivity 0.37, specificity 0.97, AUC 0.78 • Aykanli et al. (2024): GSTP1 alone sensitivity 23% and specificity 32%	• Minimally invasive • Familiar sampling • Suitable for repeated longitudinal measurement	• Low circulating tumour DNA abundance in localised disease • Dilution by non-tumour cfDNA • Differences between plasma and serum and in processing workflow • Low and inconsistent sensitivity	• Historically high specificity in some studies and meta-analyses, but contemporary performance remains inconsistent	Insufficient evidence for routine use in early detection; negative results cannot reliably exclude localised disease

Abbreviations: AUC, area under the receiver operating characteristic curve; cfDNA, cell-free DNA; csPCa, clinically significant prostate cancer; ctDNA, circulating tumour DNA; DRE, digital rectal examination; GSTP1, glutathione Stransferase Pi 1; qMSP, quantitative methylation-specific polymerase chain reaction; PSA, prostate-specific antigen

Recent population-specific evidence is broadly consistent. In a Moroccan cohort comprising 70 prostate cancer tissues and 50 controls, Mharrach et al. (2025) reported GSTP1 promoter methylation in 79% of cancers. Methylation was significantly associated with lower Gleason scores and earlier T stages, supporting its occurrence in localised disease rather than being restricted to advanced tumours. However, the relatively small cohort and use of conventional MSP limit conclusions regarding quantitative discrimination and generalisability [32].

Collectively, tissue evidence establishes GSTP1 methylation as a highly prevalent molecular characteristic of prostate adenocarcinoma. Diagnostic prevalence, however, does not necessarily translate into prognostic independence. Pidsley et al. (2024) found that higher tumour GSTP1 methylation was associated with biochemical recurrence and metastatic relapse in univariable analyses, but it did not provide independent prognostic information beyond established clinicopathological variables in multivariable models [11]. Thus, current evidence supports tissue GSTP1 principally as a marker of malignant prostate epithelium, with substantially weaker evidence that its level independently captures aggressive tumour behaviour.

### Urinary evidence: substantial potential, but pronounced methodological dependence

Urinary testing has produced some of the most clinically attractive findings because it combines a prostate-proximal specimen with non-invasive collection. Historical studies, however, reported widely varying sensitivities. Rouprêt et al. detected GSTP1 methylation in 83.2% of post-prostate-massage urine samples from men with localised cancer and found that combining GSTP1 with RASSF1A, RARβ2, and APC improved overall diagnostic discrimination [14]. These findings also illustrated the influence of collection strategy, patient selection, and analytical methodology on urinary performance.

A notable recent study of stand-alone urinary GSTP1 methylation was reported by Ji et al. (2025). Among 120 biopsy-referred patients with suspected prostate cancer, 59 had prostate cancer and 61 had benign prostatic hyperplasia. Post-DRE urinary GSTP1 methylation achieved sensitivity of 81.4%, specificity of 83.6%, accuracy of 82.5% and an area under the receiver operating characteristic curve (AUC) of 0.87. In the same cohort, PSA achieved sensitivity of 67.8%, specificity of 54.1%, accuracy of 60.8%, and an AUC of 0.67. Importantly, the difference in sensitivity between GSTP1 and PSA was not statistically significant, whereas specificity was significantly higher for urinary GSTP1 methylation [27].

In the PSA grey-zone subgroup (PSA 4–10 ng/mL), urinary GSTP1 methylation achieved sensitivity of 87.5%, specificity of 86.2%, accuracy of 86.7% and an AUC of 0.90. Combining urinary GSTP1 methylation with PSA yielded an AUC of 0.89 in the overall cohort [27].

These results identify the PSA grey zone as a plausible setting for further evaluation, but several limitations temper interpretation. The study was retrospective, single-centre, and relatively small, and participants had already been selected for biopsy based on PSA, MRI, DRE, or ultrasound abnormalities. The endpoint was biopsy-confirmed prostate cancer rather than specifically csPCa. The findings therefore describe performance in a clinically suspected, biopsy-referred population and should not be extrapolated to population screening. Moreover, comparison with PSA alone does not establish incremental value over contemporary pathways incorporating PSA density, risk calculators, mpMRI, and other adjunctive biomarkers.

### Circulating cfDNA: historically high specificity, but inconsistent contemporary performance

Blood-based evidence has generally shown a less favourable sensitivity profile. Wu et al. (2011) pooled 22 studies evaluating GSTP1 promoter methylation in bodily fluids. Among plasma, serum, and urine studies using negative-biopsy controls, pooled specificity was 0.89 (95% CI, 0.80–0.95), whereas pooled sensitivity was 0.52 (95% CI, 0.40–0.64). The authors therefore proposed GSTP1 primarily as a complement to rather than a replacement for PSA [15].

More than a decade later, Ye et al. (2023) reported an even clearer specificity–sensitivity imbalance when restricting analysis to cfDNA. Pooled sensitivity was 0.37 (95% CI, 0.23–0.53), specificity was 0.97 (95% CI, 0.88–0.99), and the summary AUC was 0.78. Substantial heterogeneity was present across diagnostic studies [21]. These findings are consistent with the biological limitations described in Sect. "From Molecular Alteration to Measurable Biomarker: Assays and Biospecimens": when methylated GSTP1 fragments are detectable in circulation, they may provide a relatively specific tumour-associated signal, but low ctDNA abundance limits the ability of a negative result to exclude localised disease.

Importantly, more recent primary evidence indicates that high circulating specificity is not universal. Aykanli et al. (2024) evaluated blood-derived cfDNA from 39 men undergoing prostate biopsy and 15 healthy controls. Among the biopsy cohort, 13 had prostate cancer, 20 benign prostatic hyperplasia, three high-grade prostatic intraepithelial neoplasia, and three atypical small acinar proliferation. GSTP1 methylation was detected in only 3 of 13 cancers but was also common in non-cancer groups, producing sensitivity of 23% and specificity of 32% for GSTP1 alone [33].

Combining GSTP1 with RASSF1 increased sensitivity and specificity to 46% and 76%, respectively, whereas requiring methylation of GSTP1, RASSF1, and RASSF2 reduced sensitivity to 8% while increasing specificity to 83% [33]. The investigators themselves noted the very small cohort as a major limitation. These findings are nevertheless instructive because they demonstrate that combining methylation markers does not necessarily improve sensitivity and specificity simultaneously; performance depends on the individual markers, assay characteristics, population, and rule used to define a positive panel.

Prognostic evidence should also be distinguished from diagnostic performance. Ye et al. reported that circulating GSTP1 hypermethylation was associated with shorter survival, with a pooled hazard ratio of 2.57 (95% CI, 1.30–5.10). However, only five studies contributed to this analysis and heterogeneity was substantial (I² = 72%) [21]. These findings should therefore not be interpreted as evidence that detecting circulating GSTP1 in an early-diagnostic population independently stratifies biologically aggressive disease.

### What has changed since the earlier meta-analyses?

The principal development since the literature captured by the 2023 meta-analysis is not a reversal of the historical performance pattern. Instead, newer studies clarify why performance varies and demonstrate that technological or methodological refinement does not uniformly translate into better clinical discrimination.

Ji et al. demonstrate substantially stronger urinary performance using a standardised post-DRE, small-volume automated workflow [27]. Visser et al., conversely, provide mechanistic evidence that partially methylated GSTP1 epialleles can compromise qMSP measurements, particularly in urine [23]. Bryzgunova et al. (2021) suggest that single-molecule interrogation of multiple CpGs may capture information lost through binary methylated/unmethylated classification [12]. Aykanli et al. (2024) add an important counterpoint: even contemporary blood-derived cfDNA testing can produce poor stand-alone GSTP1 discrimination, and combining methylation markers may increase specificity at the cost of sensitivity [33].

The systematic review by Cleanclay et al. provides a useful contemporary synthesis. Among 67 liquid-biopsy epigenetic studies published between 2015 and 2025, 14 evaluated DNA methylation. GSTP1 and RASSF1A were the most frequently reported methylation markers, but their performance as individual markers was moderate, whereas multigene approaches generally achieved greater diagnostic accuracy. Importantly, the review also highlighted the predominance of retrospective case-control studies and frequent use of healthy controls, which may inflate apparent performance compared with clinically relevant discrimination between prostate cancer and benign disease [34].

Contemporary evidence therefore does not support the conclusion that technological advances have transformed GSTP1 into a sufficiently validated stand-alone diagnostic test. Rather, GSTP1 provides a strong tumour-associated methylation signal whose observed diagnostic performance is context-dependent, varying with biospecimen, assay architecture, patient selection, comparator population, and definition of test positivity. Its most plausible future value may consequently lie in carefully defined clinical populations and in combination with complementary molecular or clinical information.

## GSTP1 alone or as part of a multiparametric strategy?

The historically high specificity but variable sensitivity of GSTP1 methylation, together with inconsistent performance across contemporary assays, raises a fundamental question regarding the optimal architecture of a GSTP1-based test. If methylated GSTP1 is absent or insufficiently abundant in a particular specimen, analytical refinement alone may not eliminate false-negative results. Combining GSTP1 with complementary molecular or clinical information therefore has a strong rationale, although increasing biomarker complexity does not necessarily improve diagnostic performance.

One approach is to combine GSTP1 with PSA. This concept is longstanding. Wu et al. (2011) proposed a complementary strategy in which PSA identifies men requiring further risk assessment and the more specific methylation signal may then refine the likelihood of malignancy [15]. Their analysis therefore positioned GSTP1 as an adjunct rather than a replacement for PSA.

Recent evidence illustrates the potential complementary use of urinary GSTP1 methylation and PSA, but does not establish a meaningful incremental benefit of combining the two markers. Ji et al. reported an AUC of 0.89 for the combined model, compared with 0.67 for PSA alone. However, urinary GSTP1 methylation alone achieved an AUC of 0.87, making the performance of the combined model numerically similar to that of GSTP1 alone [27]. In the absence of a formal comparison demonstrating a significant or clinically meaningful improvement over GSTP1 alone, these findings should not be interpreted as evidence that adding PSA provides incremental diagnostic value. More broadly, comparison with PSA alone is insufficient to establish clinical utility because contemporary biopsy decisions increasingly incorporate PSA density, validated risk calculators, mpMRI, and other adjunctive biomarkers.

A second strategy involves combining multiple methylation loci. Rouprêt et al. demonstrated this principle early: GSTP1 was the most frequently methylated individual target, while combining GSTP1, RASSF1A, RARβ2, and APC improved overall diagnostic discrimination [14]. More recently, Cleanclay et al. found that GSTP1 and RASSF1A remained the most frequently studied liquid-biopsy methylation markers, but their performance as individual markers was generally moderate, whereas multigene approaches tended to achieve greater accuracy [34].

The biological rationale is plausible. Prostate cancer is molecularly heterogeneous, and no single epigenetic alteration is guaranteed to be represented at detectable levels in every clinically relevant tumour or biospecimen. Complementary markers may capture independent aspects of malignant transformation and compensate for a weak or absent signal from an individual locus. Nevertheless, panel construction requires more than simply adding markers. Aykanli et al. (2024) found that combining GSTP1 with RASSF1 improved sensitivity and specificity relative to GSTP1 alone, whereas requiring simultaneous methylation of GSTP1, RASSF1, and RASSF2 increased specificity to 83% but reduced sensitivity to only 8% [33]. Thus, additional markers are useful only when their biological signals and the rule used to combine them contribute complementary discriminatory information.

Recent methylation-panel development also illustrates a shift towards selecting markers according to the clinical phenotype that the assay is intended to identify. Shah et al. used a multistage discovery and validation strategy to derive a 14-marker urinary methylated-DNA panel specifically evaluated for clinically significant prostate cancer. Although the panel did not contain GSTP1, it provides a useful contemporary benchmark for panel development. In a small urine pilot, sensitivity for Gleason score ≥ 7 disease was 76% at 100% specificity against healthy controls, but fell to 59% when Gleason score 6 cancers were incorporated into the reference group [35]. The latter analysis is clinically more informative because it attempts to distinguish potentially consequential disease from indolent cancer rather than simply cancer from health. The study nevertheless remains proof-of-concept and requires validation in clinically representative biopsy populations.

A third strategy is integration with non-methylation biomarkers and clinical variables. Current prostate cancer assays already combine kallikreins, RNA transcripts, genetic information, and clinical parameters. The AUA/SUO guideline lists serum assays including 4Kscore and Prostate Health Index (PHI); urine-based assays including prostate cancer antigen 3 (PCA3), MyProstateScore (MPS), SelectMDx, and ExoDx; and tissue-based ConfirmMDx for defined biopsy populations [5].

ConfirmMDx provides the most mature example of partial clinical translation involving GSTP1. Rather than relying on GSTP1 alone, it measures GSTP1, APC, and RASSF1 methylation in histologically negative biopsy tissue to detect an epigenetic field effect associated with potentially unsampled cancer. It is listed by the AUA/SUO among available biomarkers for the repeat-biopsy setting [5], whereas stand-alone urinary GSTP1 testing remains investigational. Recent reviews further emphasise that the role of ConfirmMDx is relatively narrow and must increasingly be considered alongside MRI-based diagnostic pathways [19].

The translational objective should therefore not be to demonstrate that GSTP1 is superior in isolation or that a larger panel is automatically better. Rather, development should identify the configuration in which GSTP1 provides nonredundant and clinically actionable information. This could involve GSTP1 plus PSA, a rationally selected methylation panel, a multi-analyte urine assay, or molecular information integrated with clinical and MRI-derived variables.

Accordingly, evaluation should shift from the stand-alone sensitivity and specificity of GSTP1 towards its incremental value within an existing diagnostic model. Relevant outcomes include improvements in discrimination and calibration, net clinical benefit, reduction in unnecessary biopsy, and the number of clinically significant cancers missed. A statistically higher AUC alone is insufficient if adding GSTP1 does not materially improve the clinical decision it is intended to support.

## Positioning GSTP1 within contemporary prostate cancer detection

The most important unanswered question is where GSTP1 testing would belong if an analytically robust assay became available. This requires a defined context of use: who should be tested, at which point, what decision the result should change, and what contemporary comparator it must improve upon. In 2026, that comparator is no longer PSA alone but a pathway incorporating clinical risk, PSA derivatives, biomarkers, multiparametric MRI (mpMRI), and biopsy. Except for the defined tissue-based application within ConfirmMDx, the clinical positions considered below should be regarded as proposed contexts for future evaluation rather than established indications for GSTP1 testing.

### Initial population-level screening

Current evidence does not support GSTP1 methylation as an independent population-screening test. Circulating cfDNA sensitivity remains inadequate; Ye et al. reported pooled sensitivity of 37% despite specificity of 97%. Urinary results are more encouraging but derive mainly from selected clinical cohorts rather than asymptomatic screening populations [21].

Population screening also requires more than detection of histological cancer. The objective is to identify clinically significant disease while limiting overdiagnosis and unnecessary investigation. Because GSTP1 methylation emerges early and is highly prevalent across localised cancers, a sensitive assay might detect indolent as well as consequential tumours. Its strong tissue prevalence therefore does not, by itself, justify population screening. Any screening claim would require prospective evidence of reliable detection of csPCa and acceptable downstream consequences.

### PSA grey-zone risk refinement

Risk refinement after modest PSA elevation represents a plausible context for future evaluation of GSTP1 methylation, rather than an established clinical indication. The AUA/SUO guideline states that adjunctive urine or serum biomarkers may be considered when additional risk stratification would influence biopsy decisions, typically in men with PSA levels of approximately 2.5–10 ng/mL [5]. Across secondary biomarkers included in the guideline evidence base, testing was associated with an approximately 35% reduction in biopsies, while approximately 9% of csPCa were not detected. These figures provide a contemporary benchmark for evaluating new biomarkers but should not be interpreted as expected performance for GSTP1.

Preliminary GSTP1-specific evidence in this setting comes primarily from Ji et al. (2025), who reported sensitivity of 87.5%, specificity of 86.2%, and an AUC of 0.90 for urinary GSTP1 methylation among men with PSA levels of 4–10 ng/mL. However, this finding was derived from a relatively small, retrospective, single-centre cohort of biopsy-referred patients, and the endpoint was biopsy-confirmed prostate cancer rather than csPCa, defined as Grade Group ≥ 2. The study also compared GSTP1 mainly with PSA rather than with contemporary multivariable diagnostic pathways [27]. These findings therefore support further investigation of the PSA grey zone but are insufficient to establish a clinical role for GSTP1 in this setting.

The broader pre-biopsy biomarker literature provides the appropriate standard for future evaluation. Assani et al. reviewed commercially available blood- and urine-based tests for detection of csPCa and showed that biomarker-led risk refinement may reduce unnecessary biopsies while maintaining detection of most clinically significant disease [36]. Future GSTP1 studies should therefore evaluate Grade Group ≥ 2 disease as the principal endpoint and determine whether adding GSTP1 provides incremental clinical value beyond PSA density, validated risk calculators, established secondary biomarkers, and MRI.

### Pre-biopsy triage in an MRI-based pathway

mpMRI has fundamentally changed prostate cancer diagnosis. In the AUA/SUO evidence synthesis, a Prostate Imaging Reporting and Data System (PI-RADS) 1–2 MRI had a negative predictive value of approximately 91% for Grade Group ≥ 2 cancer in biopsy-naïve men [5]. MRI therefore substantially reduces, but does not eliminate, residual risk.

Soeterik et al. (2025) provide a useful framework for positioning biomarkers around MRI. Their review found that, among men with abnormal PSA, pre-MRI risk calculators or blood- or urine-based biomarkers could avoid approximately 16–51% of MRI examinations while missing 1–16% of csPCa. In patients with equivocal MRI, particularly PI-RADS 3 lesions, biomarker- or risk-based strategies could avoid up to 72% of biopsies while missing approximately 3–13% of csPCa. Their proposed diagnostic algorithm places biomarkers alongside PSA density and risk calculators as potential pre-MRI risk-stratification tools, followed by MRI and biopsy according to residual csPCa risk [37]. These ranges should not be interpreted as expected GSTP1 performance; rather, they illustrate the pathway-level outcomes against which GSTP1 should eventually be evaluated.

Three potential positions are therefore conceivable: GSTP1 as pre-MRI triage after PSA elevation; as an adjunct after equivocal PI-RADS 3 imaging; or as a residual-risk test after a negative MRI. Assani et al. similarly note that currently available biomarkers may inform biopsy decisions in men with PI-RADS 3 findings. However, no evidence demonstrates that GSTP1 improves prediction beyond mpMRI, PSA density, and validated risk models [36]. These remain research contexts rather than established indications.

### Repeat biopsy after a previous negative result

The repeat-biopsy setting remains the most established clinical niche for a GSTP1-containing assay. After a negative biopsy, AUA/SUO guidance advises against using PSA alone as the trigger for repeat biopsy and recommends MRI when no previous prostate MRI has been performed [5].

ConfirmMDx measures GSTP1, APC, and RASSF1 methylation in histologically negative biopsy tissue to identify an epigenetic field effect associated with potentially unsampled cancer. Its inclusion among available repeat-biopsy biomarkers demonstrates that the translational barrier surrounding GSTP1 is not absolute. Translation has occurred when the marker is embedded within a defined specimen, multiplex assay, and decision context.

However, this role should not be overstated. Contemporary repeat-biopsy pathways are increasingly MRI-based, so the incremental value of tissue methylation testing must be judged against this newer standard. The broader lesson is that a narrow, decision-specific application may be more realistic than universal diagnostic use.

### Prognosis and post-treatment monitoring

GSTP1 methylation has also been investigated in prognosis, recurrence, and advanced disease. Ye et al. (2023) reported an association between circulating methylation and poorer survival [21], while Pidsley et al. found associations with biochemical recurrence and metastatic relapse that did not remain independently prognostic after multivariable adjustment [11]. These applications represent a separate translational pathway and illustrate that strong tumour association does not necessarily translate into independent prediction of subsequent clinical behaviour.

### The appropriate clinical proposition

Current evidence argues against framing GSTP1 as an “alternative to PSA.” A more defensible proposition is that GSTP1 methylation may serve as an adjunctive molecular risk signal at selected decision points where clinical, PSA-derived, and imaging information leaves sufficient residual uncertainty to influence biopsy.

Among the proposed contexts of use, post-PSA risk refinement in men with intermediate diagnostic uncertainty currently has the most direct preliminary GSTP1-specific evidence, particularly within the PSA grey zone, but remains investigational. Pre-MRI triage and refinement after PI-RADS 3 findings remain hypothetical for GSTP1, whereas the repeat-biopsy setting has achieved partial translation only through defined tissue-based multiplex testing such as ConfirmMDx. Soeterik et al. emphasise that biomarkers and MRI should be viewed as complementary rather than competing tools [37], with performance dependent on the population, local availability and cost. The potential clinical positioning of GSTP1 methylation across contemporary prostate cancer detection pathways, together with the current evidence base, relevant comparators and remaining evidence gaps, is summarised in Table 2.

**Table 2:** Potential Clinical Positioning of GSTP1 Methylation Within Contemporary Prostate Cancer Detection

Proposed clinical context	Potential role of GSTP1	GSTP1-specific evidence	Contemporary comparator	Major evidence gap	Current translational position	References
Population screening	Stand-alone early-detection test	• Strong biological signal, but circulating cfDNA sensitivity remains inadequate • Urinary evidence derives mainly from selected clinical cohorts	PSA-based risk-adapted screening	No prospective population-level evidence demonstrating detection of csPCa with acceptable downstream consequences	Not supported	• Ye et al., 2023 [21]
PSA grey-zone risk refinement	Adjunctive risk stratification after modest PSA elevation	• Preliminary urinary evidence from a retrospective single-centre study • Post-DRE urinary GSTP1 achieved an AUC of 0.90 among men with PSA 4–10 ng/mL	PSA density, validated risk calculators, established secondary biomarkers, mpMRI	Prospective validation for Grade Group ≥ 2 disease and demonstration of incremental clinical utility	Promising research context	• Wei et al., 2023 [5] • Ji et al., 2025 [27] • Assani et al., 2025 [36]
Pre-MRI triage	Refine risk after PSA elevation and potentially reduce unnecessary MRI	• No direct GSTP1-specific evidence • Broader biomarker studies support the plausibility of pre-MRI risk stratification	PSA density, risk calculators, established blood- and urine-based biomarkers	Prospective comparison of standard care with and without GSTP1	Exploratory	• Assani et al., 2025 [36] • Soeterik et al., 2025 [37]
Equivocal MRI / PI-RADS 3	Refine biopsy decisions where imaging leaves residual uncertainty	• No direct evidence that GSTP1 improves risk stratification in PI-RADS 3 lesions • Broader biomarker studies support this as a potential research context	mpMRI findings, PSA density, risk calculators	Added predictive and decision-making value beyond existing MRI-based assessment	Exploratory	• Assani et al., 2025 [36] • Soeterik et al., 2025 [37]
Negative MRI with persistent suspicion	Residual-risk assessment before biopsy	• No validated GSTP1-specific evidence demonstrates incremental value after negative MRI	PSA density, clinical risk factors, validated risk models	Prospective residual-risk studies evaluating Grade Group ≥ 2 disease	Unvalidated	• Wei et al., 2023 [5] • Soeterik et al., 2025 [37]
Previous negative biopsy	Detect molecular evidence of potentially unsampled cancer	• ConfirmMDx incorporates GSTP1, APC and RASSF1 methylation in histologically negative biopsy tissue	mpMRI and contemporary repeat-biopsy risk assessment	Incremental value within increasingly MRI-based repeat-biopsy pathways	Partially translated	• Wei et al., 2023 [5] • Robaczyńska et al., 2026 [19]
Prognosis and post-treatment monitoring	Molecular indicator of recurrence, disease burden or clinical outcome	• Associations with survival, biochemical recurrence and metastatic relapse have been reported, but independent prognostic value remains uncertain	Established clinicopathological and molecular prognostic models	Independent prognostic value and evidence that testing changes management	Investigational; separate translational pathway	• Pidsley et al., 2024 [11] • Ye et al., 2023 [21]

Note: Evidence informing pre-MRI, MRI-refinement and post-MRI strategies includes studies of biomarkers and risk tools other than GSTP1. These data are included to define the contemporary clinical context against which future GSTP1-based testing should be evaluated and should not be interpreted as evidence of validated GSTP1 performance in these settings. **Abbreviations**: APC, adenomatous polyposis coli; AUC, area under the receiver operating characteristic curve; cfDNA, cell-free DNA; csPCa, clinically significant prostate cancer; DRE, digital rectal examination; GSTP1, glutathione S-transferase Pi 1; mpMRI, multiparametric magnetic resonance imaging; MRI, magnetic resonance imaging; PCa, prostate cancer; PI-RADS, Prostate Imaging Reporting and Data System; PSA, prostate-specific antigen; RASSF1, Ras association domain family member 1

The 2026 AUA/SUO update incorporates newer evidence on MRI, biomarkers, and initial and repeat biopsy strategies, underscoring how rapidly the comparator pathway continues to evolve [4]. Future GSTP1 trials should therefore be designed against contemporary multimodal care. Success should be judged not by whether GSTP1 detects prostate cancer, but by whether adding it reduces unnecessary MRI or biopsy while preserving detection of clinically significant disease.

## Barriers to clinical translation of GSTP1 methylation

The persistence of a translational gap despite more than two decades of investigation is unlikely to have a single explanation. GSTP1 illustrates how a biologically robust cancer-associated alteration may fail to progress into routine clinical use when preanalytical standardisation, analytical validity, clinical validation, and demonstration of utility develop asynchronously. The principal barriers are therefore best considered as interconnected limitations across the biomarker-development pathway rather than as isolated technical problems [25].

### Preanalytical and biospecimen variability

The quantity and composition of measurable GSTP1-methylated DNA depend strongly on the biospecimen and how it is collected and processed. Urinary studies vary in the use of spontaneous, first-void, post-DRE, or post-prostate-massage specimens, whereas blood-based studies differ in plasma or serum preparation, processing intervals, storage conditions, and DNA extraction. These differences are particularly important because prostate-derived DNA is variably represented in urine and circulating tumour DNA may be scarce in localised disease [24, 25].

Consequently, failure to detect methylated GSTP1 in a liquid biopsy may reflect limited availability or recovery of tumour-derived DNA rather than absence of the underlying molecular alteration. Preanalytical heterogeneity therefore limits comparability between studies and complicates interpretation of apparently discordant diagnostic performance. Existing liquid-biopsy standards increasingly support harmonised reporting of key preanalytical variables, but such standardisation remains a prerequisite rather than evidence of clinical validity [38].

### Assay heterogeneity and threshold instability

Studies described as measuring “GSTP1 methylation” do not necessarily interrogate equivalent molecular states. Differences in CpG selection, primer and probe placement, conversion chemistry, analytical platform, normalisation, and positivity criteria can alter whether heterogeneous methylated molecules are detected. Earlier meta-analyses identified assay and primer-location heterogeneity as important sources of variation, while more recent work has demonstrated that partially methylated epialleles can influence quantitative measurements according to the CpGs incorporated within the assay design [15, 23].

This problem extends to interpretation thresholds. Cut-offs or multivariable algorithms optimised within small development cohorts may produce optimistic performance and may not transport reliably to independent populations. Bryzgunova et al., for example, reported promising discrimination using combinations of locus-specific methylation features but acknowledged the potential for overfitting [12]. Analytical reproducibility therefore requires not only a defined molecular target but also a prespecified assay architecture and interpretation rule that can be applied independently of the dataset in which it was developed.

### Clinically unrepresentative study populations

Many biomarker studies demonstrate biological discrimination under conditions that do not reproduce the clinical setting in which testing would ultimately be used. Comparisons between confirmed cancer cases and healthy volunteers may yield favourable estimates of diagnostic performance but do not reflect the more difficult distinction between cancer and benign conditions among men already being considered for further investigation.

Cleanclay et al. (2026) identified the predominance of retrospective case-control designs and healthy-control comparisons as important limitations in the prostate cancer epigenetic literature [34]. Clinically relevant evaluation instead requires populations resembling the intended context of use, such as men with elevated PSA, benign prostatic hyperplasia, equivocal imaging, or previous negative biopsy. Without such populations, strong discrimination may establish biological validity without demonstrating clinically useful diagnostic separation.

### Mismatch between biomarker and clinical endpoints

A further limitation is the continued use of any histologically detected prostate cancer as the primary outcome in many studies. Contemporary diagnostic pathways increasingly prioritise csPCa, generally defined as Grade Group ≥ 2, because detection of indolent disease may increase biopsy and treatment without providing equivalent clinical benefit.

This distinction is particularly relevant to GSTP1, as promoter hypermethylation occurs relatively early during prostate carcinogenesis and may therefore indicate malignant tissue without reliably distinguishing biologically consequential from indolent disease. Shah et al. (2024), although evaluating a different urinary methylation panel, demonstrated that diagnostic performance changed when low-grade cancers were incorporated into the comparator population [35]. Thus, high accuracy for detection of any prostate cancer cannot be assumed to translate into effective risk stratification for csPCa.

### Limited external validation within an evolving standard of care

Much of the GSTP1 literature remains based on relatively small or retrospective cohorts with limited independent replication. Meta-analytic evidence has highlighted heterogeneity in patient characteristics and insufficient external validation, while recent encouraging urinary findings also derive from a retrospective single-centre cohort [21, 27]. These limitations restrict confidence that reported performance will generalise to clinically representative populations.

At the same time, the diagnostic pathway against which GSTP1 must be evaluated has changed substantially. PSA density, validated risk calculators, multiparametric MRI, MRI-targeted biopsy, and established secondary biomarkers now contribute to biopsy decisions. Demonstrating superiority to PSA alone therefore no longer establishes clinical relevance. A GSTP1-based test must ultimately provide information beyond the contemporary diagnostic pathway at the decision point for which it is intended [5].

### Clinical utility and implementation remain unproven

The most consequential remaining barrier is the distinction between diagnostic accuracy and clinical utility. Sensitivity, specificity, odds ratios, or AUC quantify association or discrimination but do not establish that using a biomarker improves patient management. Translational frameworks therefore distinguish analytical validity and clinical validity from clinical utility, which requires evidence that testing provides meaningful benefit beyond existing approaches [20, 25].

For GSTP1, evidence that the molecular alteration exists, can be measured, and can distinguish selected cancer populations is substantially stronger than evidence that testing changes a contemporary clinical decision. Relatively few studies have examined whether GSTP1-guided strategies reduce unnecessary biopsy or MRI, preserve detection of csPCa, alter management, or provide sufficient practical and economic value to justify implementation. This imbalance between well-established molecular validity and uncertain decision-level benefit is central to explaining why GSTP1 promoter hypermethylation remains incompletely translated into routine prostate cancer detection.

## Evidence priorities for clinical translation of GSTP1 methylation

**Fig. 1:**
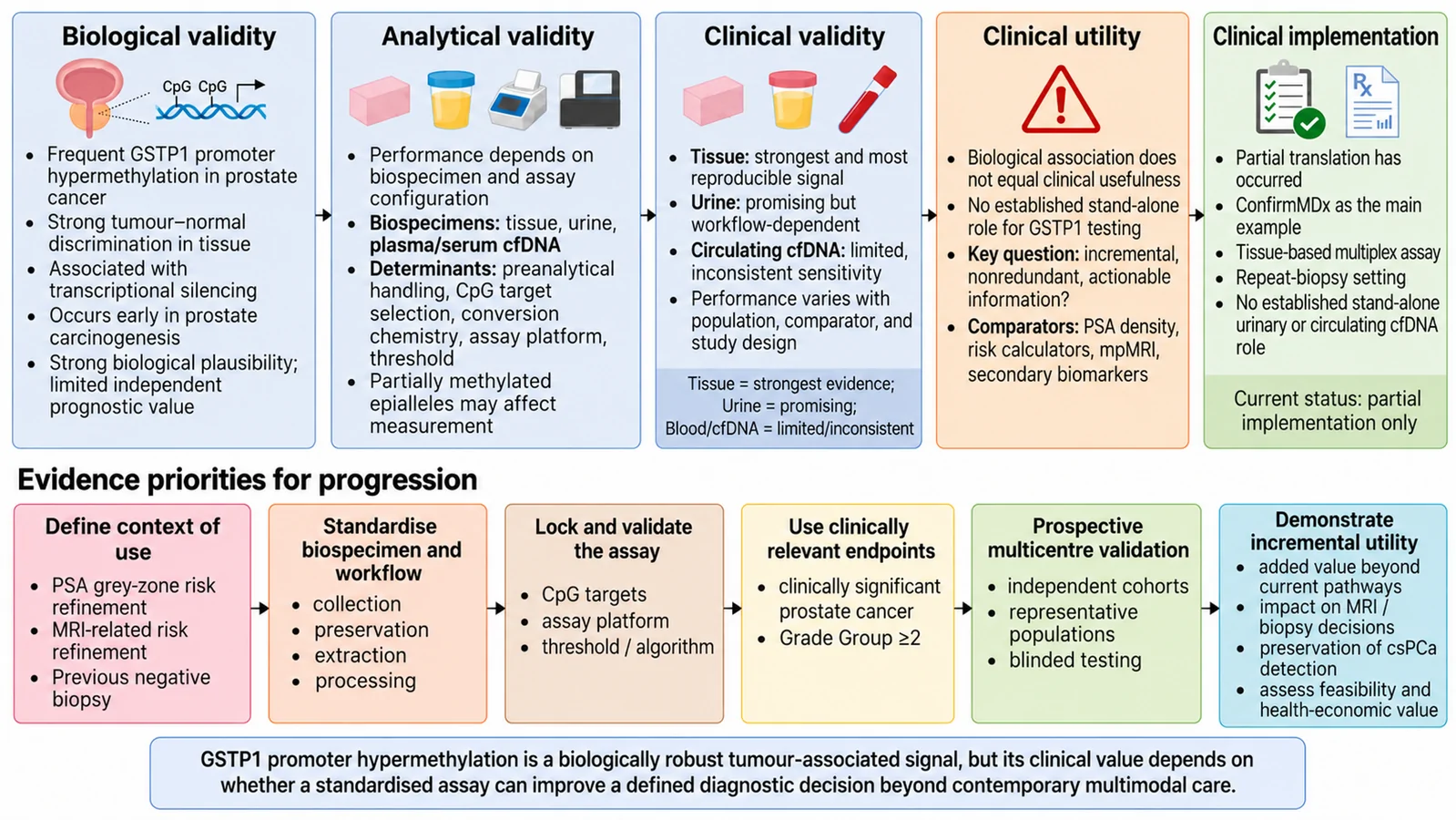
Translational pathway for GSTP1 promoter hypermethylation from biological validity to clinical implementation. GSTP1 promoter hypermethylation has strong biological validity in prostate cancer, but translation into routine clinical use depends on the analytical characteristics of the assay, biospecimen-specific clinical validity, demonstration of incremental clinical utility and feasibility of implementation. Tissue provides the strongest and most reproducible signal, whereas urinary testing remains promising but workflow-dependent and circulating cell-free DNA shows limited and inconsistent sensitivity. Partial clinical translation has occurred through tissue-based multiplex testing such as ConfirmMDx, while stand-alone urinary and circulating assays remain investigational. The lower pathway summarises the principal evidence priorities required for further translation, including definition of the intended context of use, standardisation of biospecimen and analytical workflows, assay validation, clinically relevant endpoints, prospective multicentre validation and demonstration of incremental clinical utility. cfDNA, cell-free DNA; csPCa, clinically significant prostate cancer; GSTP1, glutathione S-transferase pi 1; mpMRI, multiparametric magnetic resonance imaging; PSA, prostate-specific antigen

The next generation of GSTP1 research should move beyond repeated demonstrations that methylation is more frequent in prostate cancer than in benign tissue. The priority is to determine whether a standardised GSTP1-based assay provides reproducible, clinically meaningful information at a defined decision point within contemporary diagnostic pathways. The proposed translational pathway from biological validity to clinical implementation, together with the principal evidence priorities required for progression, is summarised in Fig. 1.

### Define the intended context of use

Studies should specify who will be tested, when testing will occur, and what decision the result is intended to change. Plausible settings include modest PSA elevation before initial biopsy, persistent suspicion after negative or equivocal MRI, and selected men with a previous negative biopsy. Among these proposed settings, the PSA grey-zone population currently has the most direct preliminary GSTP1-specific evidence, derived primarily from the retrospective single-centre study by Ji et al. [27]. This evidence is hypothesis-generating rather than sufficient to establish a clinical indication. Defining the context of use before validation is essential because the appropriate study population, comparator, performance threshold, and consequences of false-positive and false-negative results depend on the clinical decision being addressed.

### Standardise and analytically validate the complete measurement system

The biospecimen and analytical workflow should be prespecified before clinical validation. For urine- and blood-based assays, this includes standardised collection, preservation, processing, storage, DNA extraction, and input requirements appropriate to the intended clinical setting [24, 38]. The molecular assay should likewise define the CpG targets, conversion chemistry, primers or probes, normalisation method, analytical platform, and positivity rule.

Analytical validation should establish limit of detection, precision, reproducibility, stability, interference, and interlaboratory agreement, with the assay threshold or algorithm locked before external clinical testing. Consensus recommendations for cfDNA assay validation provide a useful framework for this process, particularly for blood-based applications [39]. The purpose is not simply to standardise GSTP1 as a target, but to ensure that the complete measurement system is reproducible across laboratories and populations.

### Use clinically relevant endpoints and contemporary comparators

Future studies should evaluate GSTP1 against the information already available at the intended decision point rather than against PSA alone. Relevant comparators may include age, family history, PSA density, validated risk calculators, established secondary biomarkers, prior biopsy status, and multiparametric MRI, depending on the proposed context of use.

For pre-biopsy applications, csPCa, generally defined as Grade Group ≥ 2, should be the principal diagnostic endpoint. Detection of any prostate cancer may remain a secondary outcome but is insufficient to demonstrate clinical value because GSTP1 methylation occurs early and may not reliably distinguish indolent from consequential disease. Evaluation should therefore focus on whether adding GSTP1 improves prediction of csPCa beyond contemporary standard care rather than whether the marker achieves a favourable stand-alone AUC.

### Require prospective, multicentre external validation

Promising performance should be confirmed prospectively in independent, multicentre cohorts representative of the intended clinical population. Validation should incorporate clinically realistic disease prevalence, demographic and ethnic diversity, prespecified analyses, and laboratory testing blinded to imaging and biopsy outcomes. Discovery, threshold derivation, and validation datasets should remain separate to reduce optimism arising from model development and evaluation within related cohorts.

Reporting should also provide sufficient preanalytical and analytical detail to permit reproducibility and comparison across studies. This is particularly important because incomplete reporting of collection, processing, analytical sensitivity, and specificity remains common in cfDNA biomarker research [39]. External validation should therefore assess both clinical performance and reproducibility of the predefined assay.

### Demonstrate incremental clinical utility

Diagnostic accuracy alone should not determine whether GSTP1 testing progresses towards clinical implementation. Future studies should quantify whether adding GSTP1 to an existing diagnostic pathway improves discrimination, calibration, risk classification, or decision-curve net benefit. More importantly, evaluation should include clinically interpretable outcomes such as biopsies or MRI examinations avoided, changes in biopsy recommendations, and Grade Group ≥ 2 cancers missed.

The relevant question is therefore not whether GSTP1 performs well as an isolated biomarker, but whether its addition produces nonredundant information that changes a clinical decision without an unacceptable loss of csPCa detection. A statistically higher AUC alone would be insufficient to establish such utility.

### Evaluate pathway-level feasibility and implementation

The definitive evaluation should compare diagnostic pathways rather than isolated tests, for example contemporary standard care versus standard care supplemented by a prespecified GSTP1 assay. Such studies would establish whether testing changes management while maintaining acceptable detection of csPCa.

Implementation should also consider turnaround time, assay failure, sample stability, laboratory availability, quality assurance, interlaboratory reproducibility, and cost. If clinical utility is demonstrated, health-economic evaluation should assess whether any reduction in MRI, biopsy, complications, or downstream procedures justifies the additional testing burden and cost.

Clinical translation should therefore proceed sequentially from a predefined context of use and standardised assay, through analytical validation and prospective external validation, to demonstration of incremental clinical utility and, where appropriate, pathway-level and health-economic value. Development should not proceed to implementation if GSTP1 fails to provide clinically meaningful information beyond existing diagnostic strategies.

## Conclusions

GSTP1 promoter hypermethylation is one of the most consistently documented tumour-associated molecular alterations in prostate cancer, yet its strong biological validity has not translated into a clearly established stand-alone role in contemporary detection. Tissue-based testing has achieved partial clinical translation within the multiplex ConfirmMDx assay, whereas urinary and circulating cfDNA approaches remain investigational because performance varies with biospecimen, assay configuration, study population and clinical endpoint. Current evidence therefore supports GSTP1 principally as a biologically robust molecular signal rather than as an independently validated diagnostic test.

Future clinical value will depend on whether a standardised GSTP1-based assay can provide reproducible, nonredundant and clinically actionable information beyond contemporary risk assessment, established biomarkers and MRI at a clearly defined decision point. The key question is therefore no longer whether GSTP1 methylation can detect prostate cancer, but whether measuring it improves decisions while preserving detection of clinically significant disease.

## Data Availability

No datasets were generated or analysed during the current study.
